# Effects of multiple injections on the efficacy and cytotoxicity of folate-targeted magnetite nanoparticles as theranostic agents for MRI detection and magnetic hyperthermia therapy of tumor cells

**DOI:** 10.1038/s41598-020-58605-3

**Published:** 2020-02-03

**Authors:** Meysam Soleymani, Solmaz Khalighfard, Saeed Khodayari, Hamid Khodayari, Mohammad Reza Kalhori, Mahmoud Reza Hadjighassem, Zhila Shaterabadi, Ali Mohammad Alizadeh

**Affiliations:** 1 Department of Chemical Engineering, Faculty of Engineering, Arak University, Arak, 38156-88349 Iran; 2 Brain and Spinal Cord Injury research center, Neuroscience Institute, Tehran University of Medical Sciences, Tehran, Iran; 3 Cancer Research Center, Tehran University of Medical Sciences, Tehran, Iran; 4 Department of Biology, Islamic Azad University, Science and Research Branch, Tehran, Iran; 5 Electrophysiology Research Center, Neuroscience Institute, Tehran University of Medical Sciences, Tehran, Iran; 6 Medical Biology Research Center, Health Technology Institute, Kermanshah University of Medical Sciences, Kermanshah, Iran; 7 Department of Physics, Arak University, Arak, 38156-88349 Iran; 8 Breast Disease Research Center, Tehran University of Medical Sciences, Tehran, Iran

**Keywords:** Biotechnology, Nanoparticles

## Abstract

Folate-targeted iron oxide nanoparticles (FA@Fe_3_O_4_ NPs) were prepared by a one-pot hydrothermal method and then used as cancer theranostic agents by combining magnetic resonance imaging (MRI) and magnetic hyperthermia therapy (MHT). Crystal structure, morphology, magnetic properties, surface functional group, and heating efficacy of the synthesized nanoparticles were characterized by XRD, TEM, VSM, FTIR, and hyperthermia analyses. The results indicated that the crystal structure, magnetic properties, and heating efficacy of the magnetite nanoparticles were improved by hydrothermal treatment. Toxicity of the prepared NPs was assessed *in vitro* and *in vivo* on the mammary cells and BALB/c mice, respectively. The results of the *in vitro* toxicity analysis showed that the FA@Fe_3_O_4_ NPs are relatively safe even at high concentrations of the NPs up to 1000 µg mL^−1^. Also, the targetability of the FA@Fe_3_O_4_ NPs for the detection of folate over-expressed cancer cells was evaluated in an animal model of breast tumor using MRI analysis. It was observed that T_2_-weighted magnetic resonance signal intensity was decreased with the three-time injection of the FA@Fe_3_O_4_ NPs with 24 h interval at a safe dose (50 mg kg^−1^), indicating the accumulation and retention of the NPs within the tumor tissues. Moreover, the therapeutic efficacy of the MHT using the FA@Fe_3_O_4_ NPs was evaluated *in vivo* in breast tumor-bearing mice. Hyperthermia treatment was carried out under a safe alternating magnetic field permissible for magnetic hyperthermia treatment (f = 150 kHz, H = 12.5 mT). The therapeutic effects of the MHT were evaluated by monitoring the tumor volume during the treatment period. The results showed that the mice in the control group experienced an almost 3.5-fold increase in the tumor volume during 15 days, while, the mice in the MHT group had a mild increase in the tumor volume (1.8-fold) within the same period (P < 0.05). These outcomes give promise that FA@Fe_3_O_4_ NPs can be used as theranostic agents for the MRI and MHT applications.

## Introduction

In recent years, magnetic nanoparticles (MNPs) have attracted considerable attention due to their potential application in the pharmaceutical and medicine fields such as magnetic hyperthermia therapy (MHT)^1,2^, drug delivery systems^3–5^, magnetic resonance imaging (MRI)^6,7^, gene therapy^8^, cell labeling^9^, and immunoassay^10^.

MHT has been investigated *in vivo* to treat several types of cancers, including breast, lung, brain, head and neck, prostate, pancreatic, and liver^11–18^. The principle of this method is based on the fact that cancerous cells are more sensitive to temperature rather than normal cells when the temperature is about 42–45 °C. In the MHT, magnetic nanoparticles must be introduced into the tumor tissue via systemic or direct injection and then subsequent exposure of the tumor tissue to high-frequency magnetic field results in heat generation by the MNPs via hysteresis losses, which can damage or kill cancer cells^19,20^. The heating efficiency of the MNPs under an alternating magnetic field is measured in terms of specific absorption rates (SAR). The value of this parameter is crucial for the hyperthermia application of MNPs and must be maximized because the higher SAR value leads to the smaller dose of the nanoparticles that must be injected into the body. The effects of particle size, shape, composition, and surface modification of the MNPs on the SAR value have been extensively studied by many researchers^21–23^. It has been found that MNPs in the single-domain ferromagnetic state with an open hysteresis loop can produce maximum heat under a safe alternating magnetic field^24^. Besides the appropriate size of MNPs for use in MHT, the effectiveness of this process depends upon the delivery of a sufficient dose of MNPs into the tumor tissue. The low dosage of MNPs that is accommodated into the tumor region cannot produce adequate heat in the tumor tissue, leading to the negligible or low efficacy of the treatment. Therefore, the accumulation of MNPs with appropriate size and concentration in the tumor tissue can affect the effectiveness of the MHT.

MNPs have also been used as MRI contrast agents for cancer diagnosis owing to their high r_2_ relaxivity^6,7^. In a similar manner to the MHT, the major obstacles limiting the clinical application of the MNPs as a T_2_-weighted contrast agent for MRI of tumors are the nonspecific accumulation and low concentration of the nanoparticles in the tumor tissue, causing no strong signal can be detected by the tumor tissue. One approach to enhance the accumulation of the nanoparticles in the tumor tissue is the incorporation of the tumor-specific targeting molecule on the surface of MNPs, resulting in specific uptake of the nanoparticles by cancer cells. Moreover, the tumor-targeted nanoparticles offer the advantages to decrease the side effects of the nanoparticles on normal cells. One of the favored candidates for active targeting of the MNPs is folic acid (FA), a kind of vitamin B complex, which has a high affinity to the folate receptor. Folate receptors are expressed at the relatively low levels in normal cells, but they are over-expressed in the surface of several cancer cells, including the ovary, breast, colon, lung, and brain^25^. Another approach that increases the number of nanoparticles in the tumor region is the successive injections of the targeted-nanoparticles at a safe dose, which can help the accumulation of the nanoparticles in the tumor tissue due to the enhanced permeability and retention (EPR) effects.

In the present work, dextran-coated Fe_3_O_4_ nanoparticles were synthesized using a one-pot hydrothermal method at 160 °C to produce stable and biocompatible magnetite nanoparticles with high heating efficacy for the MHT. Then, the folic acid was conjugated to the surface of the prepared nanoparticles via an esterification reaction. The prepared samples were thoroughly analyzed by different characterization analyses such as XRD, FTIR, TEM, and VSM. The *in vitro* and *in vivo* toxicity of the prepared nanoparticles were evaluated by the administration of different doses of the nanoparticles to the mammary cancer cells and BALB/c mice, and the cell viability and hematological/blood chemistry parameters were monitored. Moreover, the potential of the FA@Fe_3_O_4_ NPs to target the folate receptor cancer cells, as well as the effect of multiple injections on the accumulation of nanoparticles in the tumor tissue, was investigated by the MRI technique. Furthermore, the therapeutic efficacy of the MHT using the prepared nanoparticles was studied in breast tumor-bearing mice. The output of this study could be especially useful for improving the active-targeted MNPs as a single theranostic agent for combining MRI and MHT for the detection and treatment of cancer.

## Results and Discussion

**Figure 1:**
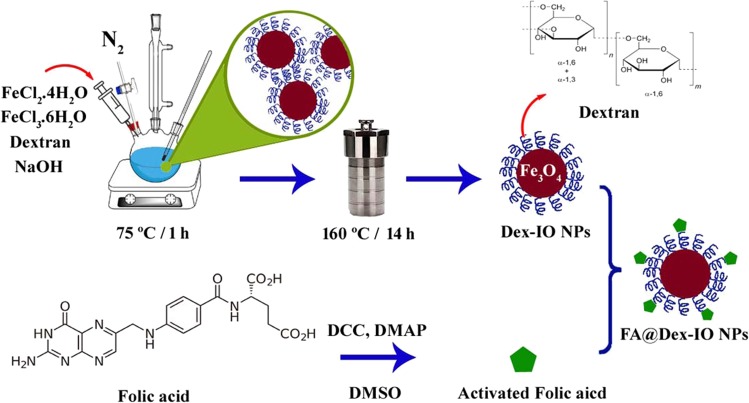
Synthesis process for the preparation of FA@Fe_3_O_4_ NPs.

The Fe_3_O_4_ NPs can be synthesized by the different types of chemical methods^20,26,27^. Nanoparticles without any surface modification are not stable in the physiological media and readily aggregate. Therefore, the surface of the Fe_3_O_4_ NPs should be modified with a suitable coating that can enhance the stability of the nanoparticles and minimize their aggregation under the physiological conditions. In this study, we synthesized the dextran-coated Fe_3_O_4_ NP by *in situ* co-precipitation of the ferric and ferrous cations in the presence of the dextran molecules followed by the hydrothermal post-synthesis to increase the size, crystallinity, and purity of the synthesized sample. Finally, folic acid as a targeting moiety was conjugated to the hydroxyl groups of the dextran on the surface of Fe_3_O_4_ NPs by esterification reaction. The whole synthesis process is schematically shown in Fig. 1.

**Figure 2:**
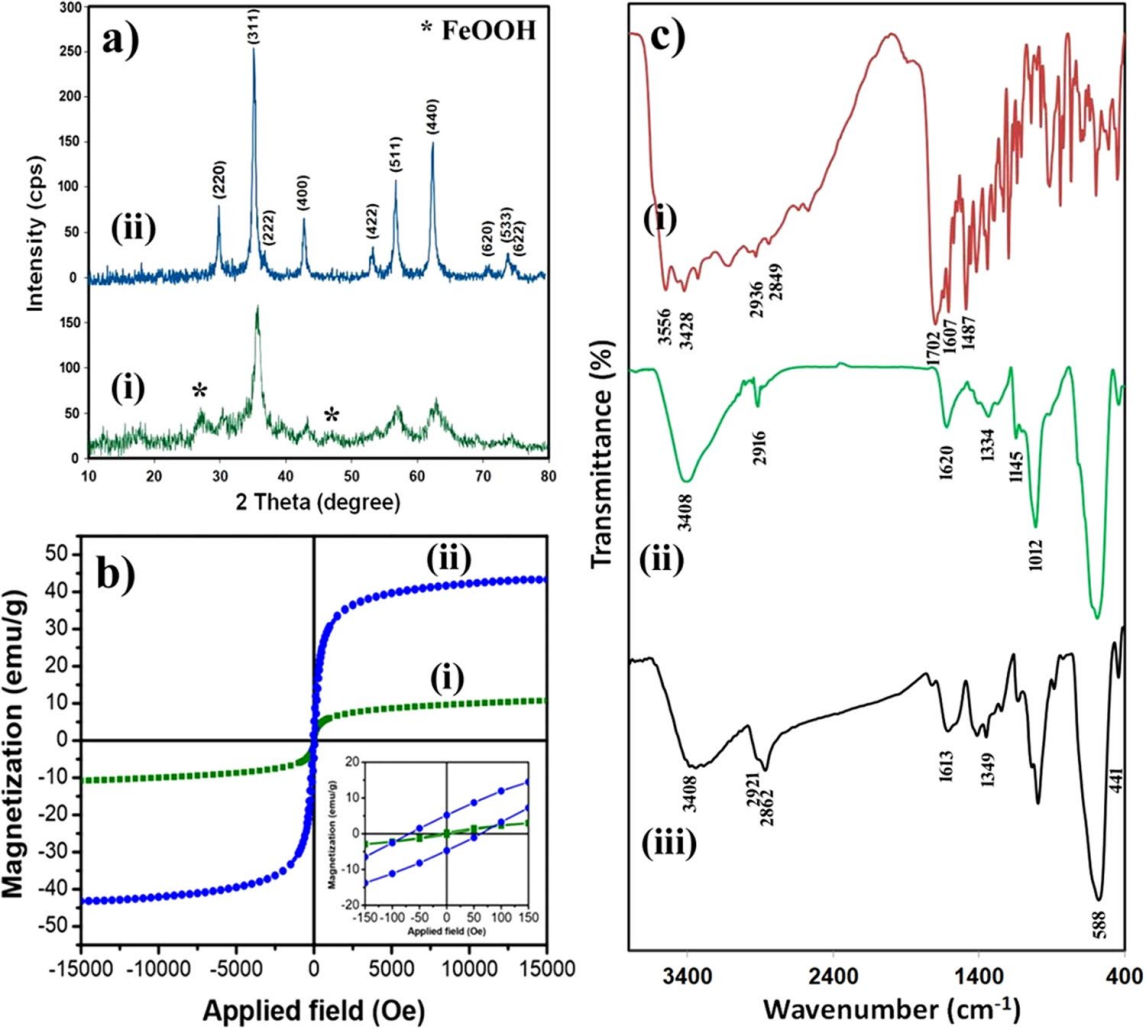
(**a**) XRD spectra of (i) sample A, and (ii) sample B, (**b**) Magnetization curves of (i) sample A, and (ii) sample B, the inset shows the magnified hysteresis loop of both samples, (**c**) FTIR spectra of (i) folic acid, (ii) dextran-coated Fe_3_O_4_ NPs, and (iii) FA@Fe_3_O_4_ NPs.

The crystal structure of the dextran-coated Fe_3_O_4_ NPs before (sample A) and after the hydrothermal treatment (sample B) was investigated by XRD analysis and the results are shown in Fig. 2a. In the XRD spectrum of sample A [Fig. 2a (i)], some impurity peaks related to the FeOOH phase (JCPDS 44-1416), the intermediate species created during the formation of the Fe_3_O_4_ NPs, were observed. On the other hand, in the XRD pattern of sample B [Fig. 2a (ii)], all reflection peaks can be indexed to a cubic structure of the Fe_3_O_4_ NPs (space group: Fd-3m) with characteristic peaks of (220), (311), (400), (422), (511), (440), (620), and (533) by referring to the JCPDS Card No. 75-0449. As can be seen, after the hydrothermal treatment, the intensity of the characteristic peaks of the Fe_3_O_4_ NPs was increased along with disappearing peaks belong to the FeOOH species.

It has been shown that the size, crystallinity, and the saturation magnetization (Ms) of magnetic nanoparticles can be increased by the hydrothermal method^28–30^. This technique is based on the Ostwald ripening mechanism in which the larger particles grow at the expense of the smaller ones. The average crystallite size of the product before and after hydrothermal treatment was estimated using the Debye-Scherrer’s formula^31^:

1 $$D=0.9\lambda /\beta cos\theta $$

Where λ is the wavelength of Cu-Kα radiation (λ = 1.54178 Å), and θ and β represent the Bragg’s angle and the full width at half maximum (FWHM) of the considered peak. The main diffraction peaks of each sample were employed for calculation. The average crystallite size of the Fe_3_O_4_ NPs before (sample A) and after (sample B) hydrothermal treatment was calculated to be about 8.6 and 21.1 nm, respectively. As can be seen, the crystallite size of the ferrite nanoparticles was significantly increased after hydrothermal treatment. Similar results were reported by other researchers^28–30,32^.

The VSM analysis was performed on the prepared nanoparticles to evaluate the effects of the hydrothermal treatment on the magnetization behavior of samples A and B. The results of the VSM analysis have presented in Fig. 2b, and the main magnetic parameters have summarized in Table 1. The inset of Fig. 2b shows the magnified view of the central region of the hysteresis loops for both samples. As can be observed, after the hydrothermal treatment, the saturation magnetization and coercivity (Hc) of the nanoparticles were significantly increased from 10.4 to 43.0 emu g^−1^ and 11.0 to 65.0 Oe, respectively.

**Table 1:** Magnetic parameters of the samples A and B.

	Sample A	Sample B
H_c_ (Oe)	11.0	65.0
M_r_ (emu/g)	0.3	9.0
M_s_ (emu/g)	10.4	43.0

The increase of Ms can be related to the conversion of FeOOH (secondary phase observed in sample A, prepared before hydrothermal treatment) to Fe_3_O_4_ phase as well as the increase of particle size of Fe_3_O_4_ NPs during the hydrothermal treatment. In fact, by increasing the particle size of nanoparticles, the surface-to-volume ratio decreases which can result in a reduction of the surface effects such as spin disorder and dead layer on the surface, leading to an increase of the magnetization^33^. Increasing the Ms is frequently considered as a straightforward approach for improving the heat generated by the MNPs^34–36^.

It has been shown that, when the size of the MNPs increases and they depart from the superparamagnetic regime, the coercivity (Hc) increases and reaches a maximum value at the single-domain ferromagnetic state and then decreases^30^. As can be observed in Table 1, the coercivity of the Fe_3_O_4_ nanoparticles increased along with the increasing of the particle size after hydrothermal treatment. The increase of the Ms and Hc in the MNPs is desirable for the theranostic (diagnostic and therapeutic) applications such as combining the MRI and magnetic hyperthermia therapy, in which MNPs with higher Hc and Ms produce higher signal intensity in the MRI applications and also MNPs with higher Ms, shape and magnetocrystalline anisotropy and enlarged hysteresis loop area have higher dissipation heat under alternating magnetic field with enough magnitude^33,37–39^. Based on the XRD and VSM results, sample B was chosen as the theranostic agent for the MRI and MHT experiments.

Folic acid was conjugated to the abundant hydroxyl groups of the dextran-coated on the surface of nanoparticles (sample B) using the esterification reaction. The FTIR spectroscopy was used to confirm the coating of the dextran as well as the further immobilizing of the folic acid on the surface of the Fe_3_O_4_ NPs.

The FTIR spectra of the pure folic acid, dextran-coated Fe_3_O_4_ NPs, and FA@Fe_3_O_4_ NPs are shown in Fig. 2c. In the FTIR spectrum of pure folic acid [Fig. 2c (i)], the absorption bands at 3556 and 3428 cm^−1^ are related to the stretching vibration of the hydroxyl groups (-OH) and N-H bonds, respectively. The absorption bands at 2936 and 2849 cm^−1^ are assigned to the asymmetric and symmetric vibrational modes of the –CH_2_ groups. Also, the strong absorption peak that appeared at 1702 cm^−1^ is attributed to the stretching vibration of the carbonyl group (C=O) in the glutamic acid moiety of the folic acid, and the absorption peak at 1607 cm^−1^ is related to the bending vibrations of the –NH bond. Moreover, the band appeared at 1487 cm^−1^ is a characteristic absorption band of the phenyl ring^40^.

In the FTIR spectra of the dextran-coated Fe_3_O_4_ NPs [Fig. 2c (ii)] and FA@Fe_3_O_4_ NPs [Fig. 2c (iii)], two absorption bands appeared in the range of 400–900 cm^−1^ arises from metal-oxygen bonds in the spinel structure of Fe_3_O_4_ nanoparticles^41^. The strong band in the range of 500–800 cm^−1^ could be related to the stretching vibration of the Fe-O bond in tetrahedral sites (ν_1_), and the small band observed in the range of 400–500 cm^−1^ is attributed to the vibration mode related to the Fe-O bond in octahedral sites (ν_2_)^41^. Also, the broad absorption peak at 3408 cm^−1^ is a distinctive stretching vibration of the hydroxyl groups presented in the dextran. In the FTIR spectrum of the dextran-coated Fe_3_O_4_ NPs, the absorption peaks at 2916 and 1334 cm^−1^ are due to the stretching and bending vibration of the –CH_2_– groups, respectively. The peak at 1620 cm^−1^ is attributed to the bending vibrations of the adsorbed water molecules on the surface of nanoparticles. Moreover, the peaks were exhibited at 1145, and 1012 cm^−1^ correspond to the stretching vibration of the alcoholic hydroxyl group (C-OH). Together these results confirm that the dextran was successfully coated on the surface of the Fe_3_O_4_ NPs.

The FTIR spectrum of the FA@Fe_3_O_4_ NPs presents all the characteristic absorption peaks of the FA and dextran-coated Fe_3_O_4_ NPs. The bands at 2921 and 2862 cm^−1^ are related to the stretching vibration of the –CH_2_– groups in the FA and dextran structures. After conjugation of folic acid to the dextran, a new absorption peak around 1702 cm^−1^ was added to the FTIR spectrum of the dextran-coated Fe_3_O_4_ NPs, which is related to the stretching vibration of carbonyl (C=O) bond in the ester group of FA, indicating the conjugation of the FA to the hydroxyl groups of dextran^42–44^. FTIR results proved that folic acid molecules have successfully immobilized on the surface of the dextran-coated Fe_3_O_4_ NPs.

**Figure 3:**
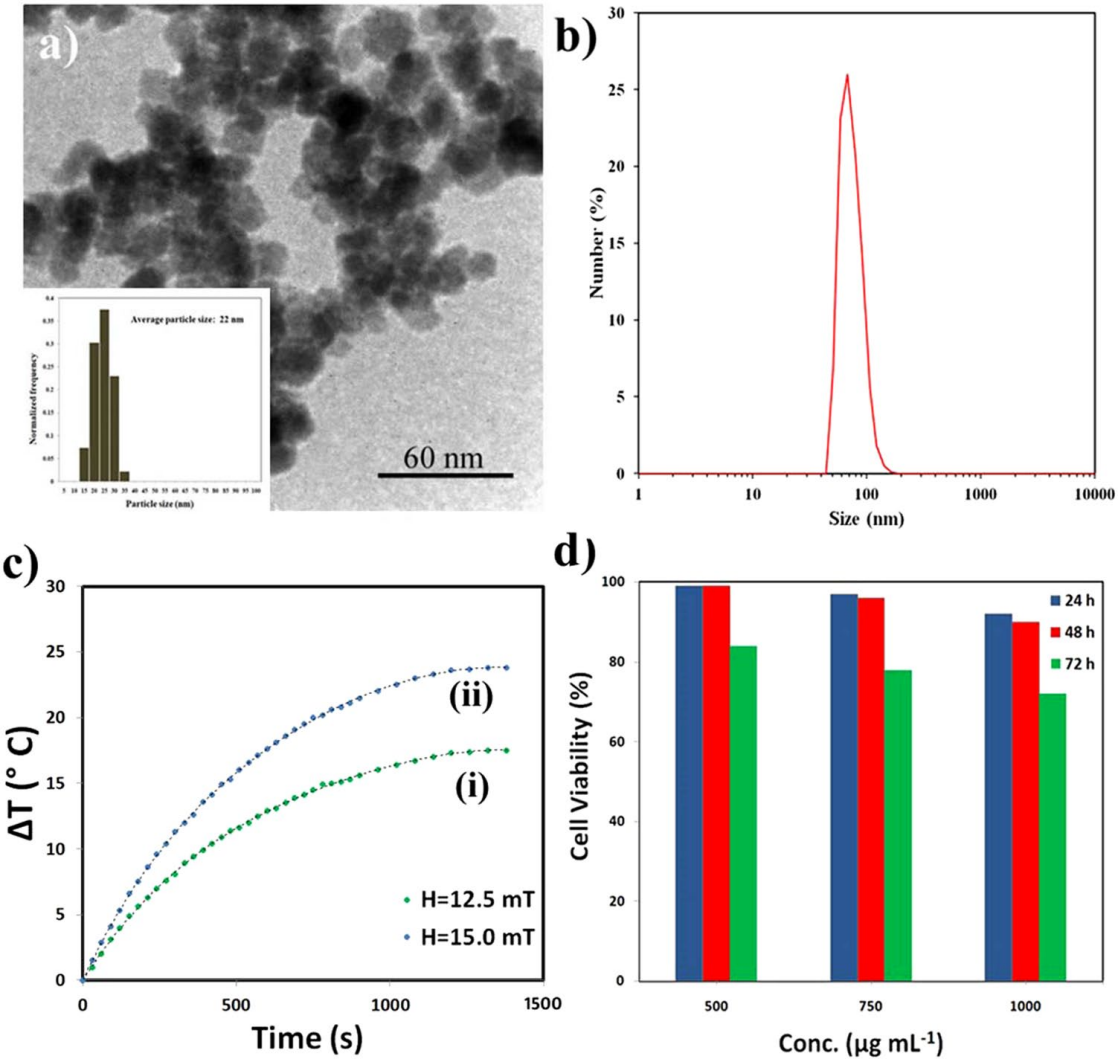
(**a**) TEM image and (**b**) particle size distribution of the FA@Fe_3_O_4_ NPs, (**c**) temperature rise vs. time curves of the magnetic suspension containing FA@Fe_3_O_4_ NPs (4 mg mL^−1^), and (**d**) cell viability of MC4*-*L2 cells after exposing to the FA@Fe_3_O_4_ NPs at different concentrations and times.

The TEM image of the FA@Fe_3_O_4_ NPs and the corresponding particle size distribution measured by more than 300 particles from several pictures are shown in Fig. 3a. As can be observed, the FA@Fe_3_O_4_ NPs have an irregular shape with an average particle size of about 22 nm. The hydrodynamic diameter of the FA@Fe_3_O_4_ NPs was also measured by DLS analysis, and the result is shown in Fig. 3b. As can be seen, this sample has a relatively sharp peak around 68.1 nm with a size distribution in the range of 50 to110 nm and mostly in the range of 60 to 90 nm. As can be observed, the size measured by the DLS analysis is larger than that obtained by TEM analysis. This issue could be due to the tendency of particles to agglomerate in the solution, water hydration, and surface charge of the nanoparticles. The impacts of the particle size on the durability of the nanoparticles in the body were investigated by several authors^45–47^. The optimum particle size of the NPs for long blood circulation time was found in the range of 10–100 nm^45–47^. Therefore, the prepared sample in the present study has suitable dimensions for biomedical applications.

The heat generation capability of the synthesized nanoparticles was investigated under two alternating magnetic fields. In this study, the safe magnetic fields with amplitudes of H = 12.5 and 15.0 mT at a fixed frequency (f = 150 kHz), which are permissible for hyperthermia therapy, were used^48,49^.

After applying the magnetic field, the temperature rise of the magnetic suspension containing FA@Fe_3_O_4_ NPs (4 mg mL^−1^) as a function of time was recorded, and the results are depicted in Fig. 3c. As can be seen, by increasing the magnetic field intensity, the higher temperature level was attained by the sample. It should be noted that the heating efficacy of the sample A (dextran-coated Fe_3_O_4_ NPs before hydrothermal treatment) was measured at the same magnetic fields, and a very low-temperature rise was observed during the experiments. This phenomenon can be related to the low magnetization and pure superparamagnetic behavior of sample A under the applied alternating magnetic field.

The SAR values calculated according to Eq. 2 for magnetic fields of H = 12.5 and 15.0 mT were 37.6 and 52.3 W g^−1^, respectively. As can be seen, the SAR value improves by increasing the magnetic field intensity. It has been found the SAR values of magnetic NPs enhance with increasing the frequency (f) and the strength of the applied magnetic field (H)^50,51^. However, for clinical application of MHT, there are two rigid and less rigid criteria for the product of the intensity (H) and frequency (f) of the applied magnetic field which called the Atkinson−Brezovich limit (H × f = 4.85 × 10^8^ Am^−1^s^−1^) and the Hergt’s limit (H × f = 5 × 10^9^ Am^−1^s^−1^), respectively^48,49^. More recently, Bellizzi and co-workers have performed a numerical study for determining the optimal frequency and magnetic field amplitude in MHT applied to the clinically relevant case of brain tumors^52^. They showed that the allowable values for H × f might be two to four times larger than the safety threshold of the Atkinson−Brezovich limit (4.85 × 10^8^ Am^−1^s^−1^) which usually considered. The possibility of using higher H × f allows us to reduce the required dosage of MNPs for an effective MHT.

The SAR values of our sample and some distinguished studies, as well as commercial Fe_3_O_4_ nanoparticles (Feridex), are presented in Table 2. As can be observed, the SAR values reported for some magnetic nanoparticles are higher than the SAR values obtained in this study (Table 2). In most cases, it can be due to the use of larger magnetic fields so that the value of H × f is far from the Hergt’s limit^48^. To better compare the heating efficiency of the FA@Fe_3_O_4_ NPs with other magnetic nanoparticles, the ILP value (normalized SAR) in each experiment was calculated, and the results are presented in Table 2. As can be observed, FA@Fe_3_O_4_ NPs with ILP value about 2.5 nHm^2^ kg^−1^ have an adequate intrinsic loss power among the other MNPs. Moreover, the ILP value of FA@Fe_3_O_4_ NPs is about 15 times higher than that of commercial Fe_3_O_4_ nanoparticles (Feridex) which have the FDA approval for biomedical applications, indicating the high potential application of the prepared sample for MHT.

**Table 2:** The magnetic field, SAR, and ILP values reported for several magnetic nanoparticles.

Magnetic nanoparticles	Frequency(kHz)	Magnetic field (kAm^−1^)	H × f (Am^−1^ s^−1^ × 10^9^)	SAR (Wg^−1^)	ILP (*nHm*^2^kg^−1^)	Reference
FA@Fe_3_O_4_	150	9.97	1.5	37.6	2.52	This study
FA@Fe_3_O_4_	150	11.96	1.8	52.3	2.44	This study
Fe_3_O_4_	300	15.0	4.1	168	2.48	^61^
MgFe_2_O_4_	700	5.0	3.5	11	0.9	^62^
CoFe_2_O_4_	370	20	7.4*	25	0.16	^63^
Zn_0.5_Ca_0.5_Fe_2_O_4_	354	10.2	3.6	14.8	0.4	^64^
CaFe_2_O_4_	354	10.2	3.6	24.5	0.66	^64^
Gd_0.02_Fe_2.98_O_4_	370	50.0	18.5*	300	0.5	^65^
Ag/Fe_3_O_4_	313	40.1	12.5*	100	0.3	^66^
γ-Fe_2_O_3_	880	7.2	6.3*	210	4.6	^67^
γ-Fe_2_O_3_	500	15.7	7.8*	106	1.4	^68^
CoFe_2_O_4_@ MnFe_2_O_4_	500	37.3	18.7*	2280	3.28	^69^
MnFe_2_O_4_@ CoFe_2_O_4_	500	37.3	18.7*	3034	4.36	^69^
Feridex	—	—	—	—	0.15	^70^

*Larger than *Hergt’s limit* (5 × 10^9^ Am^−1^s^−1^).

It is crucial to evaluate the *in vitro* toxicity of the FA@Fe_3_O_4_ NPs before employing them to MRI and MHT applications. After treating MC4L2 cells with the FA@Fe_3_O_4_ NPs at the concentrations of 250, 500, and 1000 µg mL^−1^ for 24, 48, and 72 h, the MTT assay was performed to investigate the cytotoxicity of the nanoparticles. As shown in Fig. 3d, passing the time and increasing the concentration of nanoparticles have a negative effect on the survival rate of the MC4L2 cells. Although the cell viability of the MC4L2 was decreased with increasing the concentration of the FA@Fe_3_O_4_ NPs in the medium, the concentration of 1000 μg mL^−1^ of the sample is relatively safe, so that after 72 h more than 70% cells are still alive. It can be implied that the FA@Fe_3_O_4_ NPs have no cytotoxicity even at relatively high concentrations of the nanoparticles.

**Figure 4:**
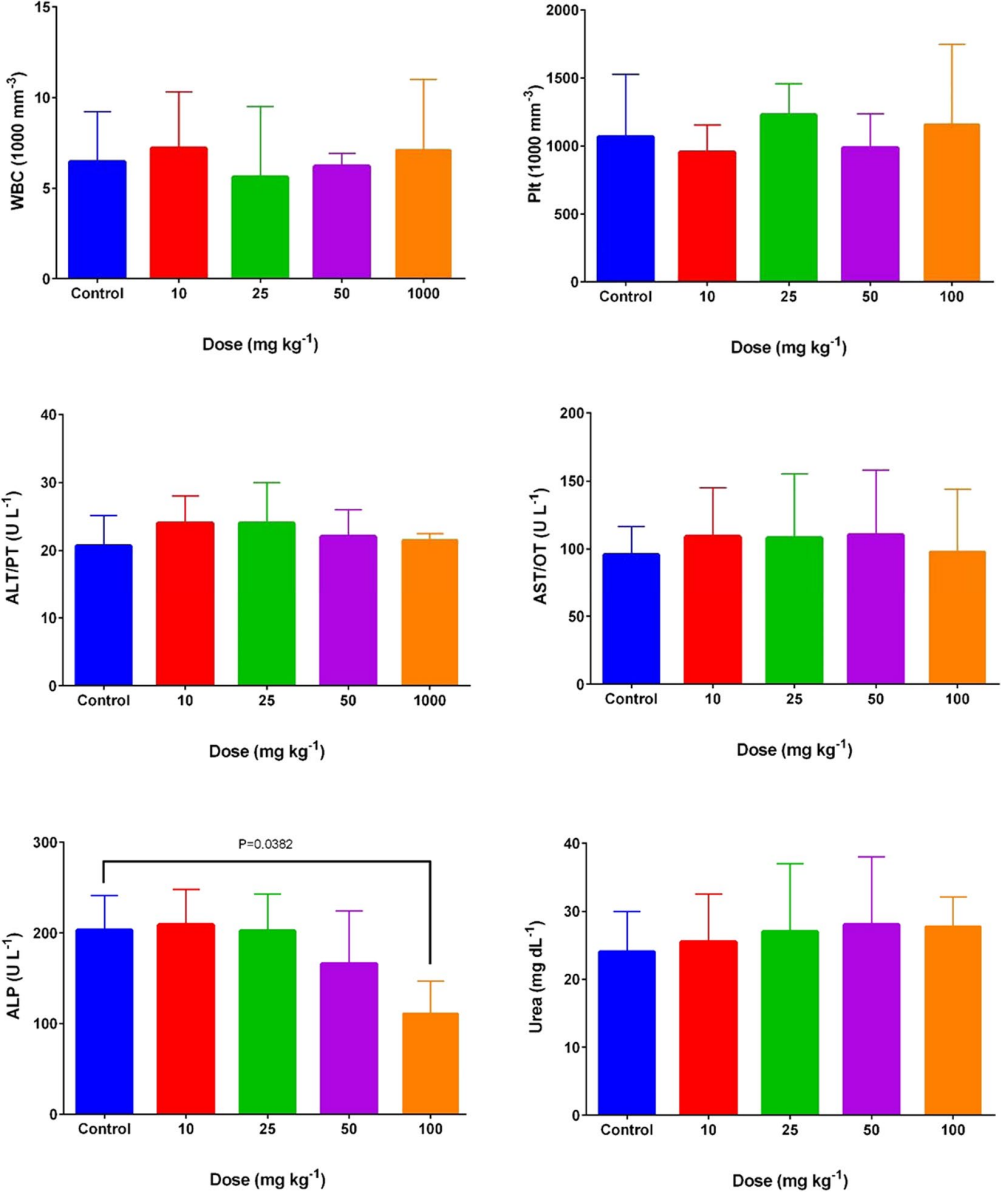
Chronic toxicity effects of FA@Fe_3_O_4_ NPs on the major hematological and blood biochemical parameters.

The primary outcomes of the *in vivo* toxicity related to the IP injection of the FA@Fe_3_O_4_ NPs at four administrative doses (10, 25, 50, and 100 mg kg^−1^) on the hematologic and clinical parameters are depicted in Fig. 4, and the full results are presented in Table 3. As can be seen, within the chronic injection of the FA@Fe_3_O_4_ NPs, no significant changes in none of the mice assayed hematologic and biochemical factors in doses of 10, 25, and 50 mg kg^−1^ were observed (P > 0.05). Only in mice treated with 100 mg kg^−1^ FA@Fe_3_O_4_ NPs, a significant decrease in the ALP hepatic enzyme has assayed compared to the control group (P < 0.05). On the other hand, no inflammatory responses were observed in all chronic toxicity groups since total white blood cells were at a reasonable range. Besides, other clinical chemistry parameters, including hemoglobin, red blood cell counts, hematocrit, and platelet counts remained within the reasonable ranges even at a high dose of FA@Fe_3_O_4_ NPs (100 mg kg^−1^). Based on the *in vitro* and *in vivo* toxicity results, a safe dose of FA@Fe_3_O_4_ NPs equal to 50 mg kg^−1^ was chosen for the MRI and MHT experiments.

**Table 3:** Hematological and blood chemical parameters for mice after different chronic doses of FA@Fe_3_O_4_ NPs.

Parameters	Control	10 (mg kg^−1^)	25 (mg kg^−1^)	50 (mg kg^−1^)	100 (mg kg^−1^)
WBC (1000/mm^3^)	6.5 ± 2.7	7.2 ± 3.1	5.6 ± 3.9	6.2 ± 0.7	7.1 ± 3.9
% Lymph	63 ± 14	60 ± 16	70.5 ± 9.1	67 ± 10.4	63.3 ± 22
RBC (Millin/mm^3^)	7.1 ± 1.4	6.9 ± 1	7.2 ± 1	6.7 ± 1.5	7.1 ± 1.6
Hgb (g/dL)	11.6 ± 1.9	11.1 ± 1	11.5 ± 1	10.2 ± 2.2	10.9 ± 2.1
HCT (%)	35.6 ± 5.2	33 ± 2.8	36.6 ± 3.2	31.9 ± 7.4	33.8 ± 8.3
MCV (FL)	49.8 ± 5.9	50.5 ± 5.6	51.2 ± 5.8	50 ± 4.7	49.1 ± 2.1
MCH (pg)	15.6 ± 2.5	15.9 ± 1.9	16.1 ± 2.3	15.9 ± 2.6	15.5 ± 2.9
MCHC (mol/L)	30.4 ± 1.7	31.6 ± 0.6	31.4 ± 0.9	31.8 ± 3	31.3 ± 2.3
PLT (1000/mm^3^)	1066 ± 462	953 ± 199	1226 ± 232	986 ± 252	1155 ± 591
BUN (mg/dL)	24 ± 6	25.5 ± 7	27 ± 10	28 ± 10	29.6 ± 4
ALP (U/L)	203 ± 38	209 ± 39	202 ± 41	166 ± 58	111 ± 36*
AST (U/L)	95.5 ± 21	109 ± 36	108 ± 47	110 ± 48	98 ± 46
ALT (U/L)	21 ± 5	24 ± 4	20 ± 10	22 ± 4	21.5 ± 1
GLU (mg/dL)	74 ± 10	65 ± 41	84.5 ± 18	85 ± 14	67 ± 19
Ca (mM/L)	3.5 ± 0.9	3.7 ± 0.3	3.7 ± 0.5	3.8 ± 0.9	4.2 ± 1
Mg (mM/L)	0.95 ± 0.1	1.2 ± 0.2	1.1 ± 0.3	1.2 ± 0.6	1.1 ± 0.5
D.Bil (mg/dL)	0.04 ± 0.03	0.06 ± 0.02	0.07 ± 0.04	0.04 ± 0.02	0.06 ± 0.02
TP (mg/dL)	1.4 ± 0.1	1.4 ± 0.2	1.3 ± 0.1	1.1 ± 0.3	1.5 ± 0.04

Values are means ± SD, and (*) indicates P < 0.05 compared to the control group. RBC = red blood cell, HCT = hematocrit, Hbg = hemoglobin, WBC = white blood cells, Plt = platelets, BUN = blood urea nitrogen, Cr = creatinine, Glu = glucose, AST = aspartate transaminase, ALT = alanine transaminase, ALP = alkaline phosphatase, Alb = albumin, T.P = total protein, B.T = Bilirubin Total, D.Bil = Direct Bilirubin. MCV = mean corpuscular volume, MCH = mean corpuscular hemoglobin, MCHC = mean corpuscular hemoglobin concentration, Ca = calcium, and Mg = magnesium.

**Figure 5:**
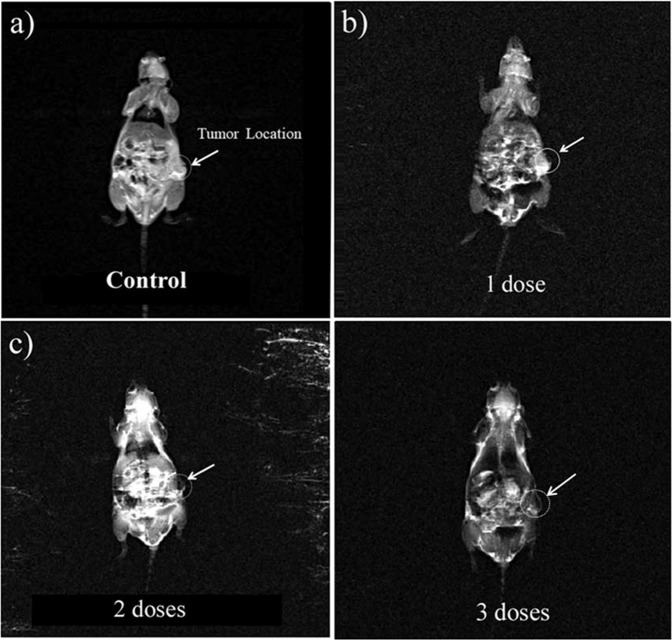
Magnetic resonance images of the mouse with a breast tumor, (**a**) mouse without injection dose (control), (**b**) mouse with one injection dose, (**c**) two injection doses, and d) three injection doses.

The potential application of the FA@Fe_3_O_4_ NPs to visualize the tumors with over-expressing folate receptors was evaluated in breast tumor-bearing mice. Figure 5 shows T_2_-weighted magnetic resonance images of mice after receiving several constant doses (50 mg kg^−1^) of FA@Fe_3_O_4_ NPs with 24 h interval. The tumor area of each mouse is shown by a white circle. The received dose in each injection was chosen based on the safe dosage, obtained in the *in vivo* toxicity experiments. The magnetic resonance image was taken after 24, 48, and 72 h of the first injection dose in each mouse. As shown in Fig. 5, the tumor tissue turned to dark in mouse with only one injection dose, suggesting the existence of the FA@Fe_3_O_4_ NPs in the tumor tissue.

**Figure 6:**
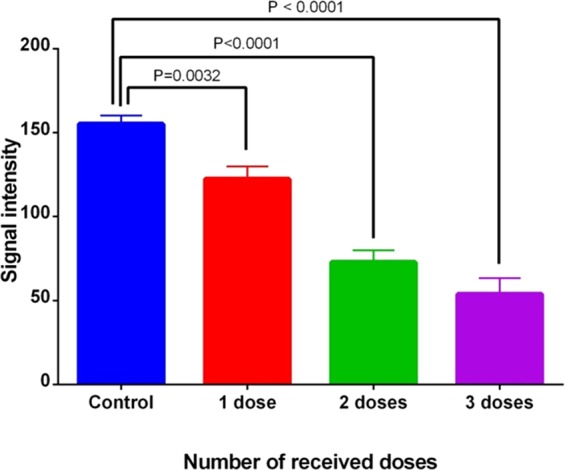
MRI signal intensity of the tumor tissue in the breast tumor-bearing mice with several receiving doses.

Furthermore, the MRI signal intensity of the tumor in all mice is shown in Fig. 6. It can be observed that the MRI signal of the tumor region was significantly decreased by repeating the IP injection of the FA@Fe_3_O_4_ NPs, so that after three injections, the lowest intensity in the tumor area was obtained, indicating the more accumulation and retention of the FA@Fe_3_O_4_ NPs in the tumor tissue. These results revealed that the FA@Fe_3_O_4_ NPs have a high potential to target the breast tumors *in vivo* and can be used as a targeted MRI contrast agent in the diagnostic research.

To give more evidence of the *in vivo* targeting capability of the FA@Fe_3_O_4_ NPs for accumulation in the tumor site, the concentration of Fe in the tumor tissues of the “control group” and “nanoparticle group” (mice in the “nanoparticles group” received three doses of the FA@Fe_3_O_4_ NPs (50 mg kg^−1^) with 24 h interval) was estimated using ICP-MS analysis. The quantitative analysis showed a significant difference (P < 0.05) of the Fe concentration in tumor tissue between control (0.7 mg _Fe_/g_Tumor_) and treated group (2.2 mg_Fe_/g_Tumor_). From the test tube experiments and *in vitro* cell hyperthermia analyses, it has been found that the minimum concentration of the iron required in the tumor tissue to produce adequate heat in the magnetic hyperthermia process must be in the range of 0.1–0.4 wt %^53–55^. According to the ICP-MS analysis results, after a three-time systemic injection of the FA@Fe_3_O_4_ NPs into the body, a sufficient concentration of Fe (0.15 wt %, calculated based on the net Fe concentration accumulated in the tumor tissue) was prepared in the tumor tissue for the MHT.

**Figure 7:**
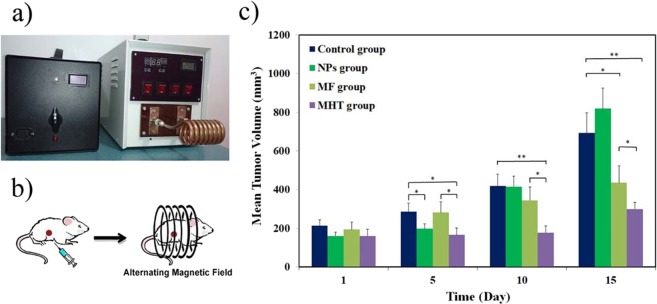
(**a**) magnetic hyperthermia unit used for *in vivo* MHT experiments, (**b**) Schematic of *in vivo* magnetic hyperthermia therapy on a mouse, and (**c**) The mean tumor volume vs days after onset of the treatment for all groups, (*) indicates P < 0.05, (**) indicates P < 0.005.

The therapeutic activity of the FA@Fe_3_O_4_ NPs was evaluated in an animal model of breast cancer. Figure 7a,b show representative images of the magnetic hyperthermia unit and a mouse lying within the designed coil. During hyperthermia experiments, neither mortality nor any significant alteration in behavior was observed in all groups during the 15 days of treatment. The tumor growth rates of all groups are depicted in Fig. 7c, A significant difference in the tumor growth between the treated mice in the MHT group and the untreated mice in the control group can be observed. In the control group (mice did not receive magnetic hyperthermia treatment) and “MF group” (mice only exposed to the alternating magnetic field several times), the tumors grew progressively. Notably, the untreated mice in the control group experienced an almost 3.5-fold increase in the tumor volume over 15 days, whereas the mice in the MHT group had a moderate increase in the tumor volume (1.8-fold) within the same period. Moreover, the tumor growth rate of the “NPs group” and “MF group” was higher than the MHT group, indicating none of the FA@Fe_3_O_4_ NPs and magnetic field solely cannot suppress the tumor growth. These results showed the effectiveness of the MHT using the FA@Fe_3_O_4_ NPs for the treatment of the breast tumor.

## Conclusions

In this study, we have successfully prepared the theranostic FA@Fe_3_O_4_ NPs as negative contrast agents for MRI as well as nanoheaters for magnetic hyperthermia treatment. The results of the MTT analysis indicated that the FA@Fe_3_O_4_ NPs were relatively safe even at high concentrations of the nanoparticles up to 1000 µg mL^−1^, satisfying one of the main requirements for biomedical applications. *In vivo* toxicity assessments revealed that the chronic toxicity of the FA@Fe_3_O_4_ NPs has appeared at the IP injection dose of 100 mg kg^−1^. Likewise, other clinical chemistry parameters, including hemoglobin, red blood cell counts, hematocrit, and platelet counts remained within the normal ranges even at this dose. The *in vivo* MRI experiments showed a significant decrease in the T_2_-weighted MR signal intensity of breast tumors by repeating the injection doses, indicating the accumulation and retention of the FA@Fe_3_O_4_ NPs in the tumor tissue. Moreover, hyperthermia treatment on an animal model of breast tumor using the FA@Fe_3_O_4_ NPs showed tumor progression could be reduced by this treatment.

## Material and Methods

### Materials

All used chemicals in this study were in the analytical grade, and they used without any further purification. Iron (III) chloride hexahydrate (FeCl_3_.6H_2_O), iron (II) chloride tetrahydrate (FeCl_2_.4H_2_O), sodium hydroxide (NaOH), dextran (Mw ≈ 10,000), folic acid (FA), *N, N’*-dicyclohexylcarbodiimide (DCC), anhydrous dimethyl sulfoxide (DMSO), 4-dimethylamino pyridine (DMAP), and MTT (3-(4,5-dimethylthiazole-2-yl)-2,5-diphenyltetrazolium bromide) powder were purchased from the Sigma-Aldrich Company. The MC4L2 cells were purchased from the Iranian Biological Resource Center (Tehran, Iran). Dulbecco’s Modified Eagle Medium (DMEM) cell culture and FBS were purchased from Scotland (Gibco, Scotland).

### The study design

The experimental studies done in this research can be categorized in four parts, including I: the preparation and characterization of the FA@Fe_3_O_4_ NPs, II: the *in vitro* and *in vivo* cytotoxicity evaluation of the FA@Fe_3_O_4_ NPs, III: the efficiency of the FA@Fe_3_O_4_ NPs for accumulation in the tumor tissues by the MRI analysis, and IV: the efficacy of the MHT by using the FA@Fe_3_O_4_ NPs for treatment of breast tumors.

### Synthesis of dextran-coated Fe_3_O_4_ nanoparticles

The dextran-coated Fe_3_O_4_ nanoparticles were synthesized by *in situ* co-precipitation of the ferrous and ferric salts in the dextran solution^20^. Briefly, the FeCl_2_·4H_2_O (1.0 mmol) and FeCl_3_·6H_2_O (2.0 mmol) were mixed in a dextran solution and then heated to 80 °C. After purging the solution with nitrogen for one hour, NaOH solution (1.0 M) was subsequently added to the mixture. Formation of the Fe_3_O_4_ NPs was indicated by the color change of the solution from the light brown to black (obtained product at this stage was named as sample A). The black suspension was then undergone to the hydrothermal treatment by transferring it to the sealed autoclave and aging at 160 °C for 14 h. The obtained product after the hydrothermal treatment named sample B.

### Synthesis of FA@Fe_3_O_4_ NPs

The conjugation of folic acid to the surface of dextran-coated Fe_3_O_4_ nanoparticles was performed by the esterification reaction between the carboxyl group of the folic acid and the hydroxyl group of the dextran^56^. Briefly, DCC (0.01 g), DMAP (0.005 g), and folic acid (0.02 g) were dissolved in the anhydrous DMSO and stirred under the N_2_ atmosphere at room temperature overnight. Then, an aqueous solution of the dextran-coated nanoparticles (5 mg mL^−1^) was added to the reaction mixture and stirred for 24 h at 80 °C under the N_2_ atmosphere in the darkness. The prepared sample was washed with the ethanol and water three times and finally suspended in the distilled water (5 mg mL^−1^) for further use.

### Characterization of FA@Fe_3_O_4_ NPs

Crystallinity and phase purity of the prepared samples were investigated by the powder X-ray diffraction (XRD, Philips, X-pert) with the Cu-K α radiation and a Ni filter (λ = 0.15418 nm). Fourier Transform Infrared (FTIR) spectroscopy analysis was carried out on a Bruker Vertex 70 spectrometer in the range of 4000-400 cm^−1^. The morphology and particle size of the samples were observed by a Transmission Electron Microscopy (TEM) with an acceleration voltage of 120 kV. The magnetic properties of the samples were measured by a vibrating sample magnetometer (VSM, Meghnatis Kavir Kashan Co., Iran) instrument at room temperature. The hydrodynamic size distribution of the prepared sample was measured by dynamic light scattering (DLS, Malvern zeta sizer-ZEN3600).

### *In vitro* toxicity of FA@Fe_3_O_4_ NPs

Through the Methyl ThiazolTetrazolium Bromide (MTT) assay, the cytotoxicity of the FA@Dex-IO NPs was evaluated on MC4-L2 cells. First, approximately 1 × 10^4^ cells per well were seeded in a 96-well plate. The cells were cultured in the DMEM medium neutralized with 10% FBS and incubated at 37 °C for 24 h in a humidified 5% CO_2_ atmosphere. Then, under treatment with various concentrations of the FA@Fe_3_O_4_ NPs (250, 500, and 1000 μg ml^−1^), the viability of cells was evaluated by the MTT method within 24, 48, and 72 h of the post-treatment.

### *In vivo* study

In this study, inbred female BALB/c mice with 6–8 weeks old (purchased from Iran Pasteur Institute) were employed. All mice were conducted within the international guidelines of the Weatherall report and also the national guidelines of the Institutional Animal Care and Use Committee (IACUC) of Tehran University of Medical Sciences.

### *In vivo* toxicity of FA@Fe_3_O_4_ NPs

To estimate the chronic toxicity dose of the FA@Fe_3_O_4_ NPs, twenty-five mice were randomly divided into five groups, with five mice in each group. The first group did not receive any injection dose of the nanoparticles and served as the control group. The second to fifth groups were intraperitoneally administrated by using FA@Fe_3_O_4_ NPs at the doses of 10, 25, 50, and 100 mg kg^−1^, respectively, for seven consecutive days. The doses were selected based on the initial acute toxicity, which showed FA@Fe_3_O_4_ NPs are safe at 100 mg kg^−1^ without any adverse effects (data not shown here). likewise, animals acutely treated with the FA@Fe_3_O_4_ NPs with doses higher than 100 mg kg^−1^ (200 and 500 mg kg^−1^) showed a significant change in the rate of ALT, ALP and AST compared to the control group (P < 0.05). One week after the last injection dose, animals were sacrificed under general anesthesia, and blood samples were taken to evaluate the hematology and clinical chemistry parameters. Red blood cells (RBC), total leukocyte count (WBC), platelets (Plt), mean platelet volume (MPV), hemoglobin (Hgb), hematocrit (Hct), mean corpuscular hemoglobin (MCH), mean cell volume (MCV), mean corpuscular hemoglobin concentration (MCHC), and lymphocytes were measured using an animal blood counter (Celltac; Nihon Kohden, Tokyo, Japan). Plasma urea nitrogen (URE), calcium (Ca), magnesium (Mg), and glucose (Glu) were determined using the CCX System (CCX WB; Nova Biomedical, USA). Plasma alkaline phosphatase (ALP), alanine transaminase (ALT), aspartate transaminase (AST), direct bilirubin (D.Bil), and total protein (TP) were also measured (Autoanalyser Model Biotecnica, BT 3500, Rome, Italy)^57^.

### Tumor transplantation

The mammary tumor stock (MC4*-*L2 cells) was aseptically separated from a breast tumor-bearing mouse, and cut into 5 mm fragments and was subcutaneously implanted in the right flank of mice to develop a breast tumor model in the BALB/c mice. After about two weeks, the mammary tumors had grown to about 150–200 mm3 and used for experimentation^58^. In all experiments, animals were anesthetized by subcutaneous injection of a 0.02 ml solution of 100 mg kg^−1^ ketamine and 10 mg kg^−1^ xylazine.

### MRI experiments

**Figure 8:**
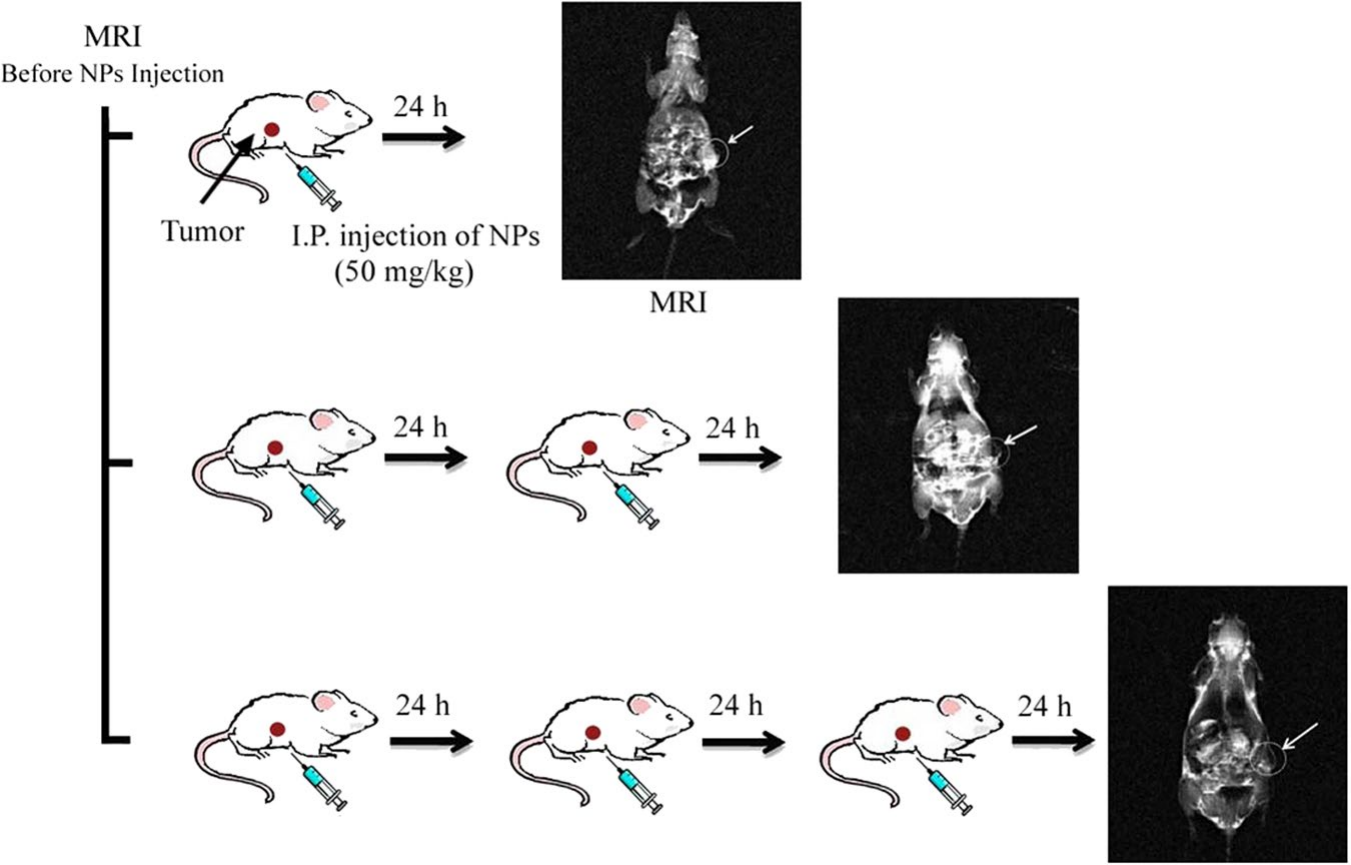
*In vivo* MRI experiments.

To evaluate the efficiency of the FA@Fe_3_O_4_ NPs for the detection and accumulation in the breast cancer tumor tissue, four breast tumor-bearing mice, labeled as 0, 1, 2, and 3, were used in the study. Mouse 0 (control) did not receive any treatment and served as the tumor signal intensity base. Mouse 1 received one dose of the intraperitoneal (IP) injection of the FA@Fe_3_O_4_ NPs (50 mg kg^−1^, 50 mg FA@Fe_3_O_4_ NPs per body weight of mouse), followed by the MRI scanning 24 h after injection. Mouse 2 received two doses of the IP injection of the FA@Fe_3_O_4_ NPs (50 mg kg^−1^) with 24 h intervals, followed by the MRI scanning after 48 h of the first injection. Mouse 3 received three doses of the IP injection of the FA@Fe_3_O_4_ NPs (50 mg kg^−1^) with 24 h intervals, followed by the MRI scanning after 72 h of the first injection. The MRI experiments were performed using a 3 T MRI scanner (Siemens, MAGNETOM Prisma). All tests were measured by a T_2_-weighted spin-echo sequence, and the parameters were set as follows: 3.5 mm slice thickness, TR = 1500, TE = 14.2, 28.4, 42.6, 56.8, 71.0, 85.2, and 99.4 ms, FOV = 180 × 180 mm, and 384 × 384 matrices. Furthermore, the MRI quantification measurements were obtained by evaluating the signal intensity of the tumor region on the T_2_-weighted MR images. The MRI experiments are schematically shown in Fig. 8.

### ICP-MS analysis of FA@Fe3O4 NPs

Six mice with breast tumors were randomly divided into two groups (three mice in each group) to evaluate the accumulation of the FA@Fe_3_O_4_ NPs in the breast tumor tissues. The groups were designated as (i) control and (ii) nanoparticles group. Mice in the control group did not receive any treatment and served as the base concentration of Fe in the tumor tissue. Mice in the nanoparticles group received three doses of the IP injection of the FA@Fe_3_O_4_ NPs (50 mg kg^−1^) with 24 h interval. Mice were then euthanized 24 h after the last injection, and their tumors were harvested. The concentration of Fe in the tumor tissues was directly analyzed by using inductively coupled plasma mass spectrometry (ICP-MS)^59^.

### Magnetic Hyperthermia experiments

The heating efficiency of the FA@Fe_3_O_4_ NPs was evaluated under safe alternating magnetic fields at a constant frequency of 150 kHz and different amplitudes (12.5 and 15.0 mT). To this end, one ml of magnetic suspension (4 mg_*Fe*304_*mL*^−1^) was inserted into an insulated microtube and then placed at the center of the induction coil. It should be noted that the organic content of FA@Fe_3_O_4_ NPs, measured by thermogravimetric analysis (TGA), was about 37%wt. After applying a certain magnetic field, the temperature rise of the magnetic suspension was recorded versus time. The specific absorption rate (SAR) of the sample was calculated according to the following equation^20^:

2 $$SAR(W{g}^{-1})=({C}_{{suspension}}/{X}_{NP})(dT/dt)$$

where, C_suspension_ and X_NP_ are the specific heat capacity of the magnetic suspension and the weight fraction of the nanoparticles in the sample, respectively. Also, *dT*/*dt* represents the initial slope of the temperature vs time curve. The intrinsic loss power (ILP) value of the samples was determined using the following equation to evaluate the intrinsic heat induction capability of the magnetic fluids and independent of magnetic field intensity and frequency^60^:

3 $$ILP(nH{m}^{2}k{g}^{-1})=SAR/f\times {H}^{2}$$

where, *f* and *H* are the frequency and intensity of the applied magnetic field, respectively.

To perform MHT using the FA@Fe_3_O_4_ NPs, twenty-four mice with breast tumors were randomly divided into four groups (6 mice in each group) to investigate the efficacy of MHT. The groups were designated as (i) control, (ii) nanoparticles (NPs), (iii) magnetic field (MF), and (iv) magnetic hyperthermia therapy (MHT). The treatment protocol of each group, including the number of times that FA@Fe_3_O_4_ NPs were injected and the number of times that the magnetic field was applied is shown in Table 4.

**Table 4:** Treatment protocol of each group.

		Treatment days									
		1	2	3	4	5	6	7	8	9	10
**Treatment groups**	Control										
	NPs	▼	▼	▼		▼		▼		▼	
	MF				□		□		□		□
	MHT	▼	▼	▼	□	▼	□	▼	□	▼	□

▼FA@Fe_3_O_4_ NPs injection.

□Magnetic field.

As can be observed in Table 4, the mice in the “control group” were not received any injection of the nanoparticles or exposed to an alternating magnetic field. The mice in the NPs group received six IP injections of the FA@Fe_3_O_4_ NPs (50 mg kg^−1^) on days 1, 2, 3, 5, 7, and 9. The animals in the MF group were exposed four times to a safe alternating magnetic field (ƒ = 150 kHz, H = 12.5 mT) for 20 min on days 4, 6, 8, and 10 without any nanoparticle injection. In the fourth group (MHT group), The animals received six injections of the FA@Dex-IO NPs (50 mg kg^−1^) on days 1, 2, 3, 5, 7, and 9 and also four times exposing to a magnetic field (ƒ = 150 kHz, H = 12.5 mT, 20 min) on days 4, 6, 8, and 10. The mice were anesthetized by IP injection of a 0.02 ml solution of 100 mg kg^−1^ ketamine and 10 mg kg^−1^ Xylazine. The tumor volume was measured every other day by using the following equation: tumor volume (mm^3^)=tumor length (mm) × tumor width (mm) × tumor width (mm) ×π/6^4,57^.

### Statistical analysis

Data were analyzed by one-way analysis of variance (ANOVA) and two-tailed Student’s t-test using the GraphPad Prism software version 6.0. P values lower than 0.05 were considered to be statistically significant.

### Ethical approval

All experiments and procedures were performed according to the guidelines of the Declaration of Helsinki (DOH), and its later amendments or comparable ethical standards. The experimental procedures and the animal use and care protocols were approved by a review board committee of Tehran University of Medical Sciences (TUMS).
